# Reviewed article: Melo MS, Souza TTCM, Soares LM, Santos AD, Raiol T, Ribeiro A. Human papillomavirus vaccination access, coverage and dropout in the Federal District, Brazil: a time series study, 2013-2023. Epidemiol Serv Saude 2025;34:e20240006. 10.1590/S2237-96222025v34e20240006.en

**DOI:** 10.1590/S2237-96222025v34e20240006.b

**Published:** 2025-04-07

**Authors:** Maria Auxiliadora Parreiras Martins

**Affiliations:** 1 Universidade Federal de Minas Gerais, Belo Horizonte, MG, Brazil

## Peer review B

## Questionnaire

Does the manuscript contain new and significant information to justify publication? Yes

Does the Abstract (Summary) clearly and accurately describe the content of the article? Yes

Is the problem significant and concisely stated? Yes

Are the methods described comprehensively? Yes

Are the interpretations and conclusions justified by the results? Yes

Is adequate reference made to other work in the field? Yes

## Manuscript Structure

Length of article is: Adequate 

Number of tables is: Adequate

Number of figures is: Adequate

**Please state any conflict(s) of interest that you have in relation to the review of this paper (state “none” if this is not applicable).**

None

**Interest:** Good

**Quality**: Good

**Originality:** Average

**Overall:** Good

**Recommendation:** Minor Revision

## Comments

- Comentário geral sobre o manuscrito
- Segundo o manuscrito avaliado, a infecção pelo papilomavírus humano (HPV, do inglês Human PapilomaVirus) está associada a diferentes tipos de câncer, especialmente, o câncer de colo de útero que representa um problema de saúde pública em todo o Brasil. A infecção por HPV e, consequentemente, os cânceres por ele causados são preveníveis, sendo a vacinação uma importante estratégia no âmbito coletivo. Trata-se de estudo ecológico de série temporal desenvolvido para analisar a tendência do acesso, cobertura e abandonoda vacinação contra a infecção pelo HPV no Distrito Federal, no período de 2013 a 2023. O tema do estudo é relevante com potenciais contribuições para o Sistema Único de Saúde. De modo geral, a redação do texto está clara e coerente. Observa-se consistência em relação ao título, introdução/objetivo, métodos, resultados/tabelas/figuras e discussão. As referências se mostram atuais e pertinentes ao tema.
- É desejável que alguns aspectos sejam considerados para aprimoramento do manuscrito:
- Revisar o texto apresentando o significado das abreviaturas em sua primeira ocorrência noresumo/abstract/ resumén (ex. HPV, MVPA)
- Estabelecer o significado original da abreviatura HPV: papilomavírus humano (HPV, do inglês HumanPapiloma Virus)
- Página 6 - linha 30: substituir “O esquema vacinal da vacina contra o HPV” por “O esquema vacinal contrao HPV”
- Segundo os autores, no ano de 2013, “iniciativas de vacinação foram implementadas nos estados do Amazonas e Distrito Federal”. Entretanto, sua introdução no calendário nacional de vacinal ocorreu a partirde 2014, sendo apresentados dados desses anos até 2023. Abordar sobre possíveis vieses na interpretaçãodos resultados em função da diferença na oferta da vacinação
- Corrigir o a legenda da Figura 2 de modo que o período seja de 2013 a 2024, conforme apresentado na ilustração
- Na Discussão, comentar se a busca ativa vacinal seria útil como estratégia para ampliação da cobertura vacinal. Os autores discutem que nas idades maiores, há redução da taxa de vacinação. Buscar referências que possam embasar o aprofundamento da discussão sobre o papel do adolescente na vacinação. O desenvolvimento gradativo da autonomia e outros aspectos comportamentais poderiam impactar na redução da imunoprevenção nessa faixa etária? E como esses elementos poderiam ser incorporados às estratégias educativas mencionadas no texto? Seguem sugestões de referências que podem ser úteis.
- https://www.scielo.br/j/csc/a/5ZSS6fQcdC9w3pcSvRpvgGD/
- https://www.scielo.cl/scielo.php?script=sci_arttext&pid=S1726-569X2023000100091&lng=en&nrm
- =iso&tlng=en

